# Eugenics in the School

**Published:** 1921-01-08

**Authors:** 


THE PUBLIC WELL-BEING—(*continued*).

### Eugenics in the School.

We select in the recent interesting lecture delivered at a conference of educational associations, which opened last week in London, the paper read by Dr. A. F. Tredgold—the author of one of the most valuable treatises on mental deficiency in the English language—in which he discussed the question of inheritance of educability. Briefly put his theory is that each person is born with a certain capacity for mental development which cannot be exceeded. In the case of children with a low intellectual capacity much of the education at present provided is a complete waste of time and money, while, on the other hand, for those of a high intellectual capacity it does not go far enough. A practical remedy which he suggests is the supplementing of scholastic examinations by medico-psychological examination.

There is no reason to quarrel with Dr. Tredgold's proposition in its broad aspects. There is little doubt, certainly, that to some extent a child's capacity for response to instruction is affected by external influences—for instance, defects in food, clothing, sleep, over-pressure. But it is probably true to say that the real cause of differences in educability lie much deeper, and is due to inheritance. Children born and bred in practically the same environment show marked differences in educability, and Sir Francis Galton has demonstrated statistically the inheritance of general ability, showing that the chance of a son of an eminent man revealing eminent ability himself is about 500 times as great as that of the son of a man taken at random. Dr. Tredgold estimates that in 1906 there was at least one certifiable mental defective in every 248 of the normal population, and this proportion has certainly increased since then. There is, however, a still larger number of individuals who, although not certifiable, have a subnormal capacity for development. Without going into the merits of Dr. Tredgold's argument in explanation of these abnormalities, the fact of their existence is obvious, and so is their intimate relation to any system of national education. It is almost inevitably a defect of an educational system, which aims at the standardisation of teaching millions of children of widely differing types, that the children of lower capacity do not receive individual or specialised attention, unless they are found to be suffering from some definite defect or disease. An advance has undoubtedly been made in methods of discrimination, and indirectly a valuable work is being performed through the organisation of medical inspection, by means of which the root cause of mental disabilities may frequently be discovered. But it is admitted that much here might be done on physiological lines to develop perception, discrimination, comparison, judgment, and reasoning, and generally to educate mental capacity.

Dr. Tredgold takes the view that the greatest defect of the British system is that it does not take enough into account the differences of innate potentiality, and it is in consequence of the fundamental differences between children of a high and those of a low intellectual capacity that the catch-phrase "equality of opportunity" is meaningless and "pure claptrap" in the absence of any equality of ability to respond to such opportunity. What is wanted, he suggests, is not so much equality of opportunity as education adapted to individual potentiality, and if more discrimination were used, and the time and money now spent in the fruitless attempt to make silk purses out of sows' ears were to be diverted to the higher education of children of good natural ability, it would contribute enormously to the efficiency of the nation. It seems to us, then, to resolve itself very largely into a question in which close medical co-operation will be required before any such system of classification can be made an accomplished fact. We believe, if public opinion could be brought round to Dr. Tredgold's view that we should get in time not only a more efficient system of education, but a far less costly one.

